# Research on Lane-Changing Decision Making and Planning of Autonomous Vehicles Based on GCN and Multi-Segment Polynomial Curve Optimization

**DOI:** 10.3390/s24051439

**Published:** 2024-02-23

**Authors:** Fuyong Feng, Chao Wei, Botong Zhao, Yanzhi Lv, Yuanhao He

**Affiliations:** 1 School of Mechanical Engineering, Beijing Institute of Technology, Beijing 100081, China; ffymieluo@126.com (F.F.); 3120235499@bit.edu.cn (B.Z.); 3120195241@bit.edu.cn (Y.L.); 3120195249@bit.edu.cn (Y.H.); 2 China North Artificial Intelligence & Innovation Research Institute, Beijing 100072, China; 3 National Key Laboratory of Special Vehicle Design and Manufacturing Integration Technology, Beijing 100081, China

**Keywords:** autonomous vehicle, lane change, decision making, trajectory planning, graph convolutional networks, multi-segment polynomial curve

## Abstract

This paper considers the interactive effects between the ego vehicle and other vehicles in a dynamic driving environment and proposes an autonomous vehicle lane-changing behavior decision-making and trajectory planning method based on graph convolutional networks (GCNs) and multi-segment polynomial curve optimization. Firstly, hierarchical modeling is applied to the dynamic driving environment, aggregating the dynamic interaction information of driving scenes in the form of graph-structured data. Graph convolutional neural networks are employed to process interaction information and generate ego vehicle’s driving behavior decision commands. Subsequently, collision-free drivable areas are constructed based on the dynamic driving scene information. An optimization-based multi-segment polynomial curve trajectory planning method is employed to solve the optimization model, obtaining collision-free motion trajectories satisfying dynamic constraints and efficiently completing the lane-changing behavior of the vehicle. Finally, simulation and on-road vehicle experiments are conducted for the proposed method. The experimental results demonstrate that the proposed method outperforms traditional decision-making and planning methods, exhibiting good robustness, real-time performance, and strong scenario generalization capabilities.

## 1. Introduction

In recent years, autonomous vehicles have become one of the research hotspots in the field of vehicle engineering. Automatic driving systems typically consist of components such as environment perception modules, decision-making modules, trajectory planning modules, and tracking control modules [1]. Among them, the decision-making module is an important bridge connecting the perception module and the planning module which needs to fully extract the perception information of the driving environment and output the correct decision instructions. The planning module can output the safe and smooth planning trajectory by receiving the instructions of the decision module, both of which play a key role in the automatic driving system.

This paper designs a comprehensive framework for autonomous vehicle lane-changing decision making and trajectory planning. It employs graph convolutional networks (GCNs) to extract decision information based on driving environment data. The autonomous vehicle, in combination with the dynamic drivable area, utilizes an optimization-based multi-segment polynomial curve trajectory planning method to plan the optimal lane-changing trajectory, enabling autonomous lane changing in complex dynamic interactive environments. The contributions of this paper are summarized as follows:

(1) Taking position, speed, and acceleration information as input, the GCN model of the driving environment is constructed, and then a vehicle decision-making method is proposed that comprehensively considers the interaction between autonomous vehicle and driving environment information, and better reflects the influence of other vehicle information on lane change decisions of the autonomous vehicle.
(2) A method for dynamically constructing convex polygon drivable areas is proposed based on the information of the ego vehicle and surrounding vehicles’ positions, velocities, and accelerations. This provides collision-free optimization space for subsequent trajectory planning, improving planning efficiency.
(3) An optimization-based multi-segment polynomial curve trajectory planning method is introduced. It divides a complete trajectory into equally timed segments of polynomial curves, each with different optimization objective functions. This targeted optimization of motion parameters for each segment enhances the tracking accuracy of the trajectory planning algorithm.
(4) Through simulation and on-road vehicle experiments, the feasibility of the proposed method is verified, and the performance is good, being superior to the existing decision-making and planning methods.

The overall structure of the paper is as follows. Section 2 reviews relevant methods in the field of autonomous vehicle behavior decision making and trajectory planning. Section 3 explains the specific construction method of the behavior decision-making module based on GCN. Section 4 provides detailed insights into the optimization-based multi-segment polynomial curve trajectory planning method. Section 5 presents the results and analysis of algorithm simulations and on-road vehicle experiments. Finally, Section 7 draws conclusions.

## 2. Related Works

Decision-making and trajectory planning play a key role in autonomous driving systems, as it is an important guarantee for autonomous vehicles to make safe, efficient, and law-compliant driving decisions and drive safely in complex environments. A large number of studies have been carried out in related fields, and the research on decision-making and trajectory planning methods of autonomous vehicles is summarized in the following sections.

### 2.1. Autonomous Vehicle Decision-Making Method

As an important part of autonomous vehicles, the decision-making module plays a crucial role in its autonomous driving process. The complex interaction process between autonomous vehicles and other traffic environment information, such as obstacles, has an important impact on autonomous vehicle decision instructions. The current mainstream vehicle decision-making methods can be divided into two categories according to their principles: non-data-driven methods and data-driven methods. Non-data-driven methods include common decision-making methods such as system construction methods based on finite state machines, game theory, etc. The hierarchical finite state machine can switch the vehicle state according to the environment of the vehicle and the defined rules and can meet the state decision requirements of simple scenarios such as valet parking and fixed-point recall [2,3]. Decision algorithms based on game theory can imitate the behavior of human drivers [4]. Stackelberg game theory is applied to the lane change modeling process of autonomous vehicles to achieve human-like interaction [5]. Meanwhile, game theory is also widely used in the behavioral decision making of autonomous vehicles in driving scenarios such as on-ramp entry on highways [6]. Data-driven decision methods are mainly divided into deep learning and reinforcement learning. These include support vector machines (SVM) [7], Extreme Learning Machines (ELM) [8], reinforcement learning (RL) [9], and deep neural networks (DNNs) [10]. The convolutional neural network model is established to detect, identify, and abstract the information in the input road scene, simulate human drivers, and make human-like lane change decisions for autonomous vehicles [11]. At the same time, the SVM algorithm can also be combined with Bayesian parameter optimization to deal with lane change decision making of autonomous vehicles [12]. The sequential decision problems of lane changing and overtaking can be modeled as Markov decision processes, and reinforcement learning methods are applied to decision making [13]. Graph neural networks (GNNs) excel in handling complex traffic scenarios and have been integrated into the decision-making processes of intelligent agents [14]. In a recent study by [15], GNNs combined with Double Deep Q-learning networks demonstrated effective multi-vehicle decision making in dynamic scenarios. Moreover, GNNs are applied to unmanned aerial vehicle (UAV) decision making [16], enabling UAVs to autonomously learn visual motion control strategies for precise landings in identified areas. These findings highlight GNNs’ crucial role in enhancing scene feature extraction and shaping behavioral decision making for intelligent agents.

### 2.2. Autonomous Vehicle Trajectory Planning Method

Autonomous vehicle trajectory planning methods can be summarized into three types of methods, which are based on graph search, sampling, and numerical optimization. Among them, the Dijkstra algorithm and A* algorithm are the most representative graph search path planning algorithms. The Dijkstra algorithm can find the shortest path from the starting point to the target point [17,18]. The A-star (A*) algorithm introduces the concept of heuristic function in graph search and defines different weights. The path search efficiency is improved [19,20]. Sample-based trajectory planning algorithms are represented by Fast Exploring Random Tree (RRT) [21] and probabilistic road map (PRM) [22]. In order to solve the problem of large computation and slow convergence of traditional sampling methods [23], a trajectory planning algorithm based on Sparse-RRT* was proposed to reduce the computation amount, and the effectiveness of the trajectory planner was verified by experimental results. In addition, another variant of the RRT* algorithm, RRT* tunable bounds, is proposed [24] to achieve faster convergence to the optimal solution, and the simulation results support the improved performance. For the trajectory planning method based on numerical optimization [25], the MIQP method is used to divide lane change into multiple stages, thereby establishing collision constraints at each stage as hard constraints, and introducing safety distance to ensure the safety of trajectory planning. Werling et al. [26] used a model-based optimization method using the Frenet coordinate system (FCOS). They transformed the programming problem into a coordinate system based on arc length, which makes the programming problem easier to solve. Researchers [27,28] proposed a spline model-based method to plan vehicle travel paths to ensure the continuity of path curvature, and applied optimization technology to solve the path planning problem. The trajectory planning of autonomous vehicles is often intricately connected with behavioral decision making. Currently, numerous studies integrate trajectory planning modules with behavioral decision modules to form a comprehensive decision-making and planning framework. For instance, ref. [29] developed a hierarchical motion planning system for lane-changing scenarios. They employed artificial potential fields to choose the target lane for lane changing and utilized fifth-order B-spline curves and quadratic programming to generate the desired trajectory. Another approach by [30] combines subsampling and optimization in a layered motion planning method for urban roads. This method employs a sampling-based approach to construct a discrete model for decision making in the driving environment, while a lower-level planner determines micro-level motion trajectories based on the integrated environmental model.

### 2.3. Problems

For autonomous vehicle behavior decision-making methods, non-data-driven methods have strong interpretability and can complete established decision-making tasks according to prior rules, but they have the problem of rule completeness and cannot complete accurate decision making for the dynamic changes in driving environment outside the scope of rules. Compared with non-data-driven methods, data-driven methods can learn driving strategies from a large amount of real driving data, which are more robust to environmental changes [31] and can be applied to a wider range of scenarios. In order to facilitate the data learning of neural networks and improve the accuracy and robustness of model construction, how to describe the characteristics of various complex driving environments more accurately is the core issue of data-driven methods. For autonomous vehicle trajectory planning methods, compared with sampling and search methods, the trajectory planning method based on optimization can usually obtain better solution results, avoid complex iteration and search processes, save computing resources, and have higher algorithm efficiency. However, complex vehicle kinematics and obstacle avoidance constraints will reduce the efficiency of solving optimization problems. How to reasonably model vehicle models and collision models is one of the urgent problems in trajectory planning. Therefore, this paper adopts GCN as the construction method of a data-driven model and uses multiple pieces of information such as position, speed, and acceleration of the ego vehicle and other vehicles to build graph structure data, so as to solve the accuracy problem of environmental feature description. At the same time, a collision-free convex polygon driving area is constructed according to the dynamic information of the ego vehicle/other vehicles, and the collision-free trajectory planning can be carried out in this area to eliminate the impact of collision detection on the trajectory planning efficiency. Finally, a multi-segment polynomial curve trajectory planning method based on optimization is adopted, and different objective functions are set in different segments to achieve the planning objectives more quickly and accurately.

## 3. Decision Making Based on GCN

### 3.1. Construction of Graph-Structured Data in Dynamic Interactive Driving Scenes

**Figure 1:**
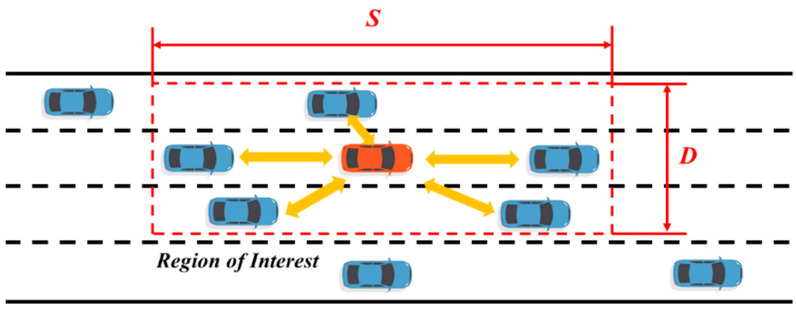
Region of interest and intervehicle connectivity strategy.

**Figure 2:**
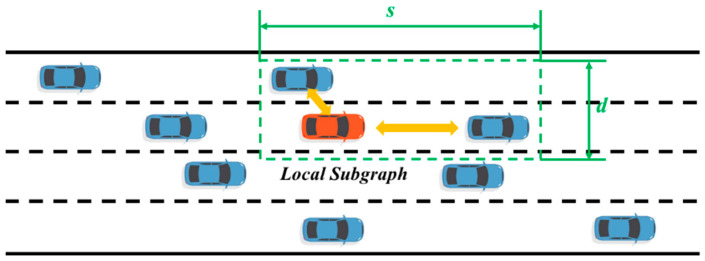
Local subgraph and intervehicle connectivity strategy.

In the typical scenario of automatic driving, the region of interest of the autonomous vehicle must be delimited first, so that the decision-making module can extract the scene information. Since real driving scenes are often complex and varied, and there are a large number of interactive driving behaviors between vehicles, it is difficult for traditional methods to effectively characterize such scenes. However, GCN models global information through the adjacency matrix method and reflects the influence of interactive behaviors in the form of connected edges, which is of great help to the characterization of interactions in dynamic scenes. Figure 1 shows the division of the regions of interest in the driving scene and the connection strategy of the interaction between vehicles. First, the regions of interest are constructed in a certain range of the front, back, left, and right of the ego vehicle according to the driving scene, the interaction diagram is constructed in the region of interest, and then the local sub-diagram is constructed according to the interaction object of the driving behavior of the self-driving car according to the specific lane change decision instruction, as shown in Figure 2. Thus, a comprehensive representation of the driving scene can be achieved from the two levels of the interest region and the local subgraph.

For GCN, the layer-by-layer propagation rules are as follows:

(1) $$H^{\left(l+1\right)}=\sigma ({\tilde{D}}^{-\frac{1}{2}}\tilde{A}{\tilde{D}}^{-\frac{1}{2}}H^{\left(l\right)}W^{\left(l\right)})$$

The vehicle is represented as a node and an adjacency matrix with *N* nodes is constructed:

(2) $$\tilde{A}=A+I_{N}$$

where $A\in {\mathbb{R}}^{N\times N}$, $I_{N}$ is the identity matrix, $\tilde{A}$ is the adjacency matrix with added self-join, $\tilde{D}$ is the degree vector of $\tilde{A}$, $W^{\left(l\right)}$ is the layer’s weight lth, σ(⋅) is the activation function, and $H^{\left(l\right)}$ is the layer’s activation matrix lth.

(3) $$H^{\left(0\right)}=X$$

X is the feature matrix of the input node, whose data are obtained by the sensor and can be expressed as:

(4) $$X=\left[x_{self},x_{other}\right]$$

In order to fully consider the influence of the status of the ego vehicle/other vehicles on decision instructions, the position, speed, and acceleration information of the ego vehicle/other vehicles are all taken into account in the process of constructing the interaction relationship between nodes in the input GCN diagram, where $x_{self}$ represents the node characteristics of the ego vehicle and $x_{other}$ represents the node characteristics of other vehicles, which are specifically expressed as follows:

(5) $$x_{self}=\left[x_{0},y_{0},v_{x},v_{y},a_{x},a_{y}\right]$$

(6) $$x_{other}=\left[x_{r},y_{r},v_{xi},v_{yi},a_{xi},a_{yi}\right]$$

where $i\in \left\{0,1,\ldots ,N-1\right\}$. The characteristics of the ego vehicle include the lateral distance $y_{0}$ relative to the center line of the initial lane and longitudinal distance $x_{0}$, as well as the lateral speed $v_{y}$ and longitudinal speed $v_{x}$ and the lateral acceleration $a_{y}$ and the longitudinal acceleration $a_{x}$. Other vehicle characteristics include the lateral distance $y_{r}$ and longitudinal distance $x_{r}$ relative to the ego vehicle, as well as the lateral speed $v_{yi}$ and the longitudinal speed $v_{xi}$ and the lateral acceleration $a_{yi}$ and the longitudinal acceleration $a_{xi}$.

For dynamic interaction scenarios, we use the connection strategy shown in Figure 1 to characterize the interaction relationship between vehicles. First, the region of interest is modeled hierarchically, and a global interaction graph containing all vehicles in the region of interest and a local subgraph containing only interaction objects of self-driving vehicles are constructed, respectively. Self-driving vehicles are connected to all vehicles in the graph through undirected edges. Since the node feature matrix contains the relative position information between vehicles, the connected edge only represents whether there is a connected relationship between two nodes through the following formula.

(7) $$a_{ij}=\left\{\begin{array}{ll} 0 & \mathrm{unconnected}\\ 1 & \;\;\;\mathrm{connect} \end{array}\right.$$

The aggregated global information is input into GCN in the form of graph structure data to output the behavior decision instructions of the autonomous vehicle, that is, the mapping relationship between the graph structure data of dynamic scenes at every moment and the optimal driving behavior instructions of the autonomous vehicle is established, and it is used in the subsequent trajectory planning module.

### 3.2. Dataset Construction and Network Details

**Figure 3:**
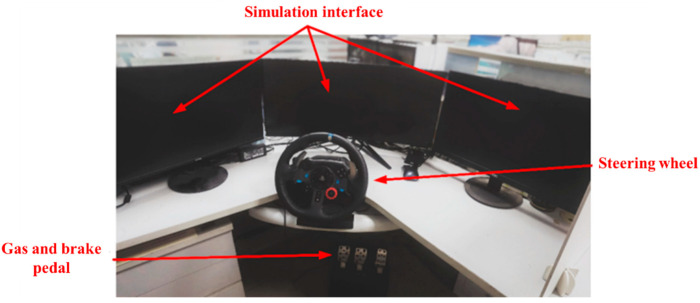
Simulation experiment driving simulator.

In this paper, real driving data are collected by CARLA [32] simulation platform to train the network of the decision module. The dynamic driving environment under different working conditions was set for each set of data collection, and the surrounding vehicles were set to automatic driving mode by using “set_autopilot(True)” in CARLA’s PythonAPI. For the ego vehicle, the driver uses the external driving simulator shown in Figure 3 to control ego vehicles, during which the relevant driving data are recorded and saved.

For driving tasks in different dynamic environments for a certain period of time, each set of data in the dataset has been realized; the total driving data have exceeded 300 sets and the total driving mileage has exceeded 60 km. Among them, each set of data contains the perception data of the ego vehicle and other vehicles. The ego vehicle’s data include the lateral speed $v_{y}$ and longitudinal speed $v_{x}$, the lateral distance $d_{y}$ relative to the center line of the lane before the lane change, and the action performed, D, that is, the behavioral decision instruction. The other vehicles’ data include the lateral distance $y_{r}$ and longitudinal distance $x_{r}$ relative to the ego vehicle, as well as the lateral speed $v_{yi}$ and longitudinal speed $v_{xi}$ and lateral acceleration $a_{yi}$ and longitudinal acceleration $a_{xi}$.

**Figure 4:**
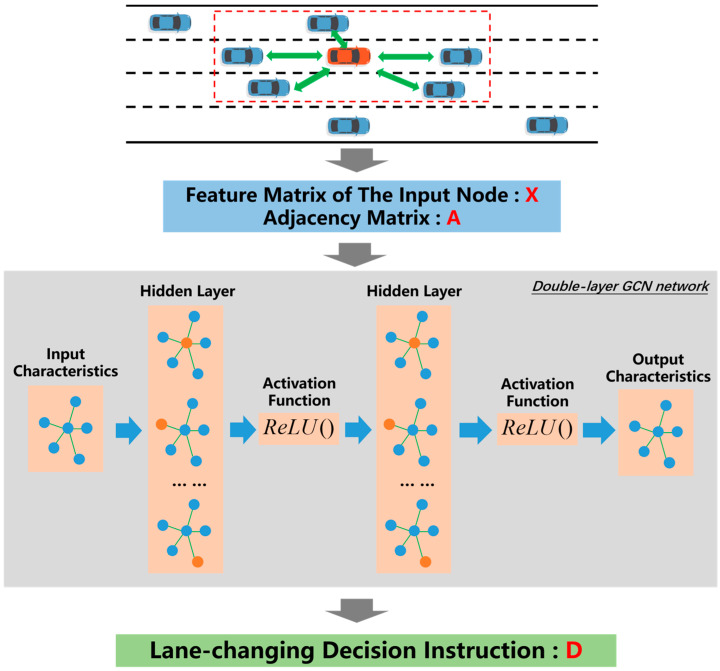
Illustration of GCN structure.

For GCN, the model in the network was trained using the Adam optimizer [33]. The initial learning rate was set to 0.001, and the learning rate was set to 0.98 by using the exponential attenuation strategy. In order to avoid the over-smoothing phenomenon caused by too many convolutional layers of graphs, this paper sets two convolutional layers, inputs the constructed adjacency matrix A and node feature matrix X into GCN, and the activation function of each layer is a linear rectification function ReLU(). The specific construction process of GCN is illustrated in Figure 4. Then, the global graph embedding is obtained by pooling the point embedding of the whole graph, and finally, the probability of each decision instruction is output by normalized exponential function Softmax(). The action decision command with the highest probability is the output, and the loss function is the Cross-Entropy Loss function CrossEntropyLoss(). The detailed GCN construction parameters are summarized in Table 1.

## 4. Construction of Drivable Area

**Figure 5:**
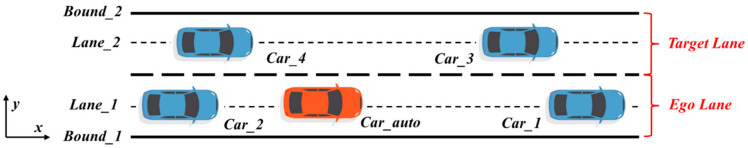
Illustration of the lane-changing scenario.

According to the local subgraph obtained by the decision-making module, the lane-changing scenario as shown in Figure 5 is defined, where Lane_1 is the current lane of the ego vehicle, Lane_2 is the target lane of the lane change, the middle is the dividing line of the two lanes, and Bound_1 and Bound_2 are the lower and upper boundaries of the road, respectively. Car_auto is an automatic driving vehicle, Car_1 and Car_2 are the front and rear vehicles of the current lane, respectively, and Car_3 and Car_4 are the front and rear vehicles of the target lane, respectively.

**Figure 6:**
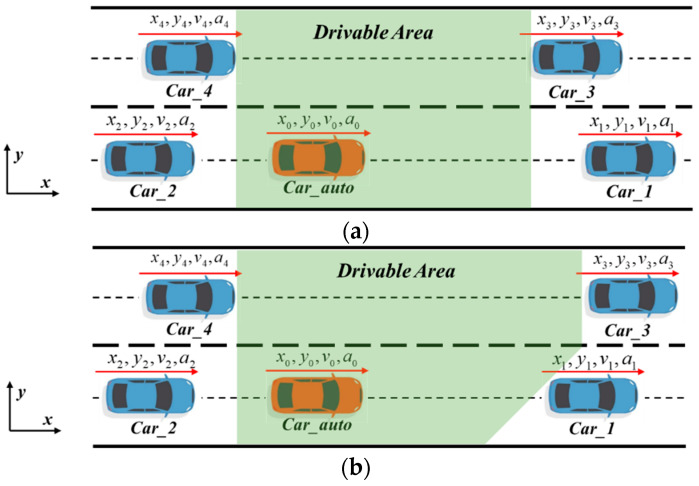
Illustration of the drivable area. (**a**) Rectangular drivable area. (**b**) Pentagonal drivable area.

In order to ensure the safety of the trajectory, the obstacle avoidance constraints of the ego vehicle and other vehicles should be considered in the trajectory planning module. Excessively complex obstacle avoidance constraints will affect the solving speed of the optimization algorithm. If the distance between the ego vehicle and the other vehicle is directly used as a constraint, the constraint is non-convex, which increases the complexity of the trajectory planning problem. If the distance between the ego vehicle and the other vehicles is taken as part of the objective function, it is impossible to guarantee that the distance between the two vehicles is greater than 0, and it is difficult to strictly guarantee that there will be no collision between the ego vehicle and other vehicles. Therefore, this paper first considers the lane boundary and the position, speed, and acceleration information of the surrounding vehicles, establishes a dynamic convex polygon drivable area without obstacles, limits the vehicle trajectory to the drivable area, and constructs a convex constraint, thus ensuring strict non-collision between the ego vehicle and other vehicles. The drivable area is shown in Figure 6.

The types of drivable areas can be divided into two categories. When the longitudinal position of the rear boundary of vehicle Car_1 is larger than the longitudinal position of the rear boundary of vehicle Car_3, a rectangular drivable area, as shown in Figure 6a, is established. When the longitudinal position of the rear boundary of vehicle V_ob1_ is smaller than the longitudinal position of the rear boundary of vehicle V_ob3_, a pentagonal drivable area, as shown in Figure 6b, is established. The rear boundary of the drivable area is determined by Car_2 and Car_4. Since the vehicles are in the forward driving state, only the front boundary of the vehicles in front of the two rear vehicles can be taken as the back boundary of the drivable area, which can reduce the complexity of constraints without affecting the lane change planning.

**Figure 7:**
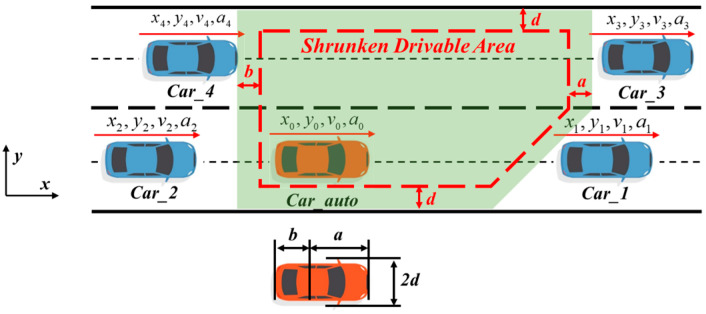
Illustration of the shrunken drivable area.

In consideration of the vehicle’s inherent volume, directly assessing whether the center of the vehicle is within the aforementioned drivable area still poses a collision risk. Therefore, this paper advocates the contraction of the convex polygon, ensuring that the vehicle strictly adheres to obstacle avoidance constraints. Assuming that the vehicle’s center of mass position is the reference point, with a distance of ‘a’ to the front end and ‘b’ to the rear end from the vehicle’s center, and a vehicle width of 2d, the drivable area is shrunk based on these parameter details. The principle is illustrated in Figure 7.

The shrunken convex polygon area can be described by:

(8) $$M(t)X(t)+n(t)\leq 0,\begin{array}{cc} & t\in [0,T] \end{array}$$

where $X(t)={\left[x(t),y(t)\right]}^{T}$, x(t) and y(t), respectively, represent the longitudinal position and lateral position of the vehicle at time *t*, and *T* represents the total duration of the planned trajectory. And the edges of the shrunken kyphotic polygon are determined by M(t) and n(t).

## 5. Trajectory Planning of Multi-Segment Polynomial Curve Based on Optimization

### 5.1. Construction of Polynomial Equation of Trajectory Curve

The complete trajectory is divided into a *k-segment* sub-trajectory. The specific number of segments *k* of this sub-trajectory is not subject to fixed constraints, and the trajectory segmentation is actually determined according to the real-time requirements of the computing power and computing time of the computing platform.

The planning time of the complete trajectory is *T*, the planning time of all sub-trajectories is the same, and the planning time corresponding to the sub-trajectory of the *i* segment is *T_i_*, and then it can be expressed as:

(9) $$T_{i}=\frac{T}{k}\begin{array}{cc} & \left(i=1,2,3\ldots k\right) \end{array}$$

For the longitudinal position of paragraph *k*, a quintic polynomial is used to describe it:

(10) $$x_{i}(t)=a_{i0}+a_{i1}t+a_{i2}t^{2}+a_{i3}t^{3}+a_{i4}t^{4}+a_{i5}t^{5}$$

Hence, the longitudinal velocity ${\dot{x}}_{i}(t)$, acceleration ${\ddot{x}}_{i}(t)$, and the rate of acceleration change ${\dddot{x}}_{i}(t)$ can be described as:

(11) $${\dot{x}}_{i}(t)=a_{i1}+2a_{i2}t+3a_{i3}t^{2}+4a_{i4}t^{3}+5a_{i5}t^{4}$$

(12) $${\ddot{x}}_{i}(t)=2a_{i2}+6a_{i3}t+12a_{i4}t^{2}+20a_{i5}t^{3}$$

(13) $${\dddot{x}}_{i}(t)=6a_{i3}+24a_{i4}t+60a_{i5}t^{2}$$

Thus, the undetermined coefficient of the longitudinal position expression of the sub-trajectory of the *i* section can be expressed as a vector $X_{ia}$, which can be described as follows:

(14) $$X_{i}={\left[a_{i0},a_{i1},a_{i2},a_{i3},a_{i4},a_{i5}\right]}^{T}$$

Using the same method, the lateral position of paragraph *k* can be obtained:

(15) $$y_{i}(t)=b_{i0}+b_{i1}t+b_{i2}t^{2}+b_{i3}t^{3}+b_{i4}t^{4}+b_{i5}t^{5}$$

And then, the lateral velocity ${\dot{y}}_{i}(t)$, acceleration ${\ddot{y}}_{i}(t)$, and the rate of acceleration change ${\dddot{y}}_{i}(t)$ can be described as:

(16) $${\dot{y}}_{i}(t)=b_{i1}+2b_{i2}t+3b_{i3}t^{2}+4b_{i4}t^{3}+5b_{i5}t^{4}$$

(17) $${\ddot{y}}_{i}(t)=2b_{i2}+6b_{i3}t+12b_{i4}t^{2}+20b_{i5}t^{3}$$

(18) $${\dddot{y}}_{i}(t)=6b_{i3}+24b_{i4}t+60b_{i5}t^{2}$$

Finally, the undetermined coefficient of the lateral position expression of the sub-trajectory of the *i* section can be expressed as a vector $X_{ib}$, which can be described as follows:

(19) $$X_{ib}={\left[b_{i0},b_{i1},b_{i2},b_{i3},b_{i4},b_{i5}\right]}^{T}$$

### 5.2. Parameter Optimization of Polynomial Trajectory Curve

In order to ensure the high real-time requirements of the trajectory planning algorithm, this paper will build a quadratic programming optimization model for trajectory planning problems, give full play to the advantages of high solving efficiency of the quadratic programming model, and optimize the lateral/longitudinal trajectory expression parameters. The standard form of the quadratic programming model is built as follows:

(20) $$\begin{array}{l} \min\;J=X^{T}QX+2q^{T}X\\ s.t.\;Gx\leq h\\ \begin{array}{c} \;\;\;\;\;\;\;\;AX=b \end{array} \end{array}$$

where $X={\left[{X_{0a}}^{T},{X_{1a}}^{T}\ldots {X_{ka}}^{T},{X_{0b}}^{T},{X_{1b}}^{T}\ldots {X_{kb}}^{T}\right]}_{1\times 12k}^{T}$. It is used as the optimization parameter of the lateral and longitudinal trajectory optimization model.

#### 5.2.1. Cost Function

In this paper, a multi-segment polynomial trajectory planning method is adopted. Different cost functions will be set for different subsegment trajectories, which can be optimized according to the specific parameters of different subsegment trajectories to obtain higher-quality trajectories.

In order to facilitate the description, the cost function is divided into a lateral cost function and longitudinal cost function. The cost function expression of the sub-trajectory in the *i* section is as follows:

(21) $$J_{ix}=J_{ix\_T}+J_{ix\_C}\begin{array}{cc} & \left(i=1,2,3\ldots k\right) \end{array}$$

(22) $$J_{iy}=J_{iy\_T}+J_{iy\_C}\begin{array}{cc} & \left(i=1,2,3\ldots k\right) \end{array}$$

where $J_{ix}$ is the cost function of the longitudinal position of the sub-trajectory in the *i* section, which includes longitudinal moving target cost $J_{ix\_T}$ and longitudinal comfort cost $J_{ix\_C}$. Similarly, $J_{iy}$ is the cost function of the lateral position of the sub-trajectory in the *i* section, which includes lateral moving target cost $J_{iy\_T}$ and lateral comfort cost $J_{iy\_C}$.

The moving target includes four parts: longitudinal target position $x_{target}$, longitudinal target speed $v_{target}$, lateral target position $y_{target}$, and lateral target speed 0. From this, we can obtain the concrete expression of the target cost of longitudinal/lateral motion:

(23) $$J_{ix\_T}=\sum_{j=0}^{j=N}\left(\omega_{ix\_pos}{\left(x_{i}(t_{j})-x_{target}\right)}^{2}+\omega_{ix\_speed}{\left({\dot{x}}_{i}(t_{j})-v_{target}\right)}^{2}\right)$$

(24) $$J_{iy\_T}=\sum_{j=0}^{j=N}\left(\omega_{iy\_pos}{\left(y(t_{l})-y_{target}\right)}^{2}+\omega_{iy\_speed}{\dot{y}}_{p}^{2}(t_{l})\right)$$

where N is the number of trajectory points of the sub-trajectory longitudinal position curve, which can be calculated by $N=T_{i}/\Delta t$; $t_{j}$ is the time corresponding to the *j-th* trajectory point, which can be calculated by $t_{j}=j\Delta t$. The weight coefficient for the cost function of the longitudinal position curve of the *i*-th segment of the sub-trajectory is denoted as $\omega_{ix\_pos}$ and $\omega_{ix\_speed}$. The weight coefficient for the cost function of the lateral position curve of the *i*-th segment of the sub-trajectory is denoted as $\omega_{iy\_pos}$ and $\omega_{iy\_speed}$.

For the comfort cost, the comfort of the trajectory is mainly related to the rate of acceleration change. Therefore, we take the sum of squares of the rate of acceleration change of the longitudinal trajectory as the longitudinal comfort cost $J_{ix\_C}$ and the sum of squares of the rate of acceleration change of the lateral trajectory as the lateral comfort cost. The weight coefficient for the cost function of the comfort cost of the *i*-th segment of the sub-trajectory is denoted as $\omega_{ix\_jerk}$ and $\omega_{iy\_jerk}$. The specific expression is as follows:

(25) $$J_{ix\_C}=\sum_{j=0}^{j=N}\left(\omega_{ix\_jerk}{\dddot{x}}_{i}^{2}(t_{j}\right))$$

(26) $$J_{iy\_C}=\sum_{j=0}^{j=N}\left(\omega_{iy\_jerk}{\dddot{y}}_{i}^{2}(t_{j}\right))$$

Using (20) and (21), the complete cost function of multi-segment polynomial parameter optimization can be expressed as:

(27) $$J=\sum_{i=0}^{i=k}\left(J_{ix}+J_{iy}\right)=\sum_{i=0}^{i=k}\left(\left(J_{ix\_T}+J_{ix\_C}\right)+\left(J_{iy\_T}+J_{iy\_C}\right)\right)$$

Using (23)–(26), the specific expression of the cost function is as follows:

(28) $$J=\sum_{i=0}^{i=k}\left(\begin{array}{l} \sum_{j=0}^{j=N}\left(\omega_{ix\_pos}{\left(x_{i}(t_{j})-x_{target}\right)}^{2}+\omega_{ix\_speed}{\left({\dot{x}}_{i}(t_{j})-v_{target}\right)}^{2}\right)+\sum_{j=0}^{j=N}\left(\omega_{ix\_jerk}{\dddot{x}}_{i}^{2}(t_{j}\right))+\\ \sum_{j=0}^{j=N}\left(\omega_{iy\_pos}{\left(y(t_{l})-y_{target}\right)}^{2}+\omega_{iy\_speed}{\dot{y}}_{p}^{2}(t_{l})\right)+\sum_{j=0}^{j=N}\left(\omega_{iy\_jerk}{\dddot{y}}_{i}^{2}(t_{j}\right) \end{array}\right)$$

The optimization priorities of different motion parameters can be modified by adjusting the size of $\omega_{ix\_pos}$, $\omega_{ix\_speed}$, $\omega_{iy\_pos}$, $\omega_{iy\_speed}$, $\omega_{ix\_jerk}$, and $\omega_{iy\_jerk}$, so as to achieve the goal of emphasizing the optimization of specific parameters in different sub-trajectories.

#### 5.2.2. Constraints

(1) Continuity constraint

In order to ensure the continuity of autonomous vehicle motion, the lateral/longitudinal motion parameters of the initial point of the output trajectory must be consistent with the motion state of the current position of the vehicle to ensure the continuity of the four motion parameters of the initial point position, speed, acceleration, and rate of acceleration change, which are specifically expressed as follows:

(29) $$\left\{\begin{array}{l} x_{1}(0)=x_{0}\\ {\dot{x}}_{1}(0)={\dot{x}}_{0}\\ {\ddot{x}}_{1}(0)={\ddot{x}}_{0}\\ {\dddot{x}}_{1}(0)={\dddot{x}}_{0}\\ y_{1}(0)=y_{0}\\ {\dot{y}}_{1}(0)={\dot{y}}_{0}\\ {\ddot{y}}_{1}(0)={\ddot{y}}_{0}\\ {\dddot{y}}_{1}(0)={\dddot{y}}_{0} \end{array}\right.$$

where $x_{1}(0)$, ${\dot{x}}_{1}(0)$, ${\ddot{x}}_{1}(0)$, and ${\dddot{x}}_{1}(0)$ are the initial longitudinal position, velocity, acceleration, and rate of acceleration change of the sub-trajectory of the first section. $x_{0}$, ${\dot{x}}_{0}$, ${\ddot{x}}_{0}$, and ${\dddot{x}}_{0}$ are the current longitudinal position, velocity, acceleration, and rate of acceleration change of the sub-trajectory. Using the same definition, the expression of the continuity constraint of the initial lateral point can be obtained, as shown in (29).

In order to make a smooth transition between k trajectories based on the optimized multi-segment polynomial curve trajectory planning method, it is necessary to ensure continuous motion parameters between subsegments, which means the motion parameters between the end point of the previous segment and the initial point of the next segment must be equal. We mainly consider the continuity of four motion parameters: position of connection point, speed, acceleration, and acceleration change rate, which are specifically expressed as:

(30) $$\left\{\begin{array}{l} x_{i}(T_{i})-x_{i+1}(0)=0\\ {\dot{x}}_{i}(T_{i})-{\dot{x}}_{i+1}(0)=0\\ {\ddot{x}}_{i}(T_{i})-{\ddot{x}}_{i+1}(0)=0\\ \begin{array}{l} {\dddot{x}}_{i}(T_{i})-{\dddot{x}}_{i+1}(0)=0\\ \begin{array}{l} y_{i}(T_{i})-y_{i+1}(0)=0\\ {\dot{y}}_{i}(T_{i})-{\dot{y}}_{i+1}(0)=0\\ {\ddot{y}}_{i}(T_{i})-{\ddot{y}}_{i+1}(0)=0\\ {\dddot{y}}_{i}(T_{i})-{\dddot{y}}_{i+1}(0)=0 \end{array} \end{array} \end{array}\right.\begin{array}{cc} & i=1,2,3\ldots \ldots k-1 \end{array}$$

(2) Security constraint

In the process of trajectory planning, it is necessary to ensure that the trajectory has no collision with the obstructed vehicle and does not exceed the road boundary range. In Section 4, safety constraints have been constructed for parameter optimization of multi-segment polynomials, and the position of the vehicle centroid is constrained in the shrunken drivable area. The collision-free trajectory can be generated by taking the centroid of the ego vehicle as the planning starting point. The influence of complex constraints on the optimization problem is reduced. The specific constraints are expressed as follows:

(31) $$M_{i}(t)X_{i}(t)+n_{i}(t)\leq 0,\begin{array}{cc} & t\in [0,T_{i}] \end{array}$$

where $X_{i}(t)={\left[x_{i}(t),y_{i}(t)\right]}^{T}$, and $x_{i}(t)$ and $y_{i}(t)$ represent the longitudinal position and lateral position of the vehicle at time *t* of the sub-trajectory of section *i*, respectively. $T_{i}$ represents the total time range of the sub-trajectory. The edges of the drivable area after contraction within the time range of the sub-trajectory of section *i* are determined by $M_{i}(t)$ and $n_{i}(t)$.

The lane change motion planning problem can be established as a standard quadratic programming problem, as mentioned in Equation (20), and the special model is shown in Equation (32). By solving the quadratic programming problem, the optimal trajectory can be obtained.

(32) $$\begin{array}{l} \min(28)\\ s.t.(29),(30),(31) \end{array}$$

## 6. Simulation and On-Road Vehicle Experiments

### 6.1. Simulation Experiment

In this paper, the proposed method is verified by simulation experiments. The experiment was carried out on the CARLA simulation platform under UBUNTU. CARLA is an open-source simulator based on Unreal Engine 4, which has realistic simulation scenes and can have various sensors set.

**Figure 8:**
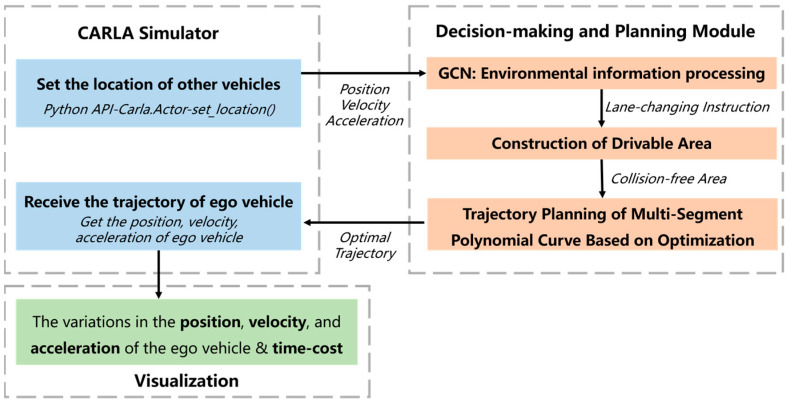
Overall framework of the simulation experiment.

In the simulation experiment, several other vehicles are first set, and each vehicle is set to the automatic driving mode using the relevant API of the simulation platform. The speed information and position information of other vehicles are obtained through the simulation platform for decision-making and planning algorithms. The driving behavior of the ego vehicle in different driving scenarios is recorded to evaluate the method proposed in this paper. In this paper, OSQP [34] was used to solve the optimization model constructed in Section 5 to obtain the optimal trajectory. Finally, relevant graphs are generated based on the simulated vehicle motion data and the corresponding computational time information. The overall framework of the simulation experiment is illustrated in Figure 8.

**Figure 9:**
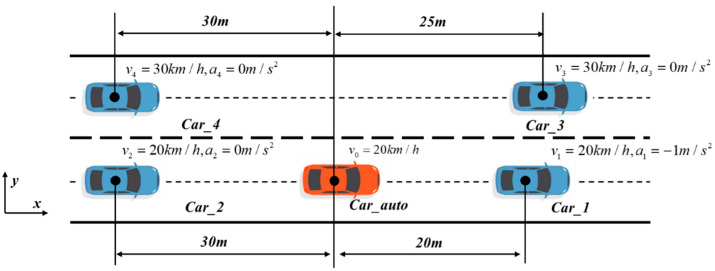
Illustration of simulation experiment lane-changing scene.

As shown in Figure 9, the ego vehicle is driving at a speed of 20 km/h in the current lane, and the speed of the front and rear vehicles in the current lane is 20 km/h. Moreover, the front vehicle in the current lane is undergoing uniform deceleration with an acceleration of −1 m/s^2^. The speed of the front and rear vehicles in the target lane is 30 km/h. The vehicle needs to change lanes from the current lane to the target lane and travel at a target speed of 30 km/h.

**Figure 10:**
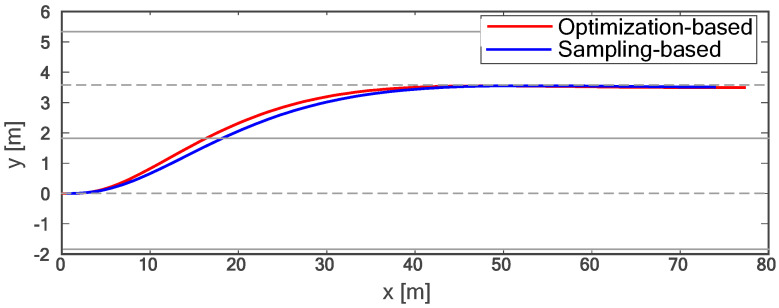
Comparison of lane-changing trajectories.

**Figure 11:**
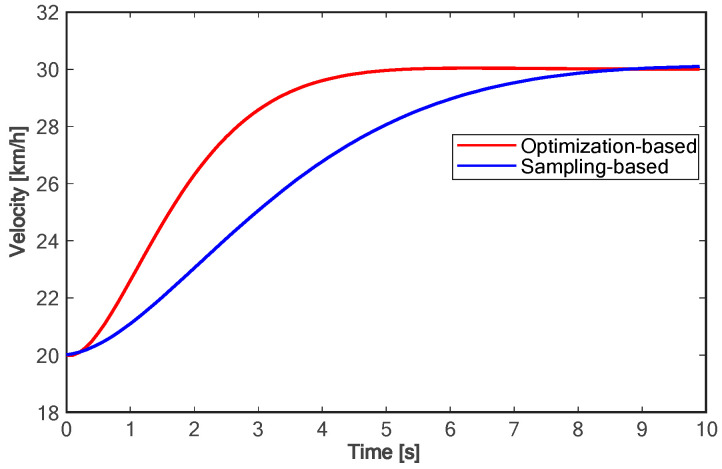
Comparison of longitudinal velocities.

**Figure 12:**
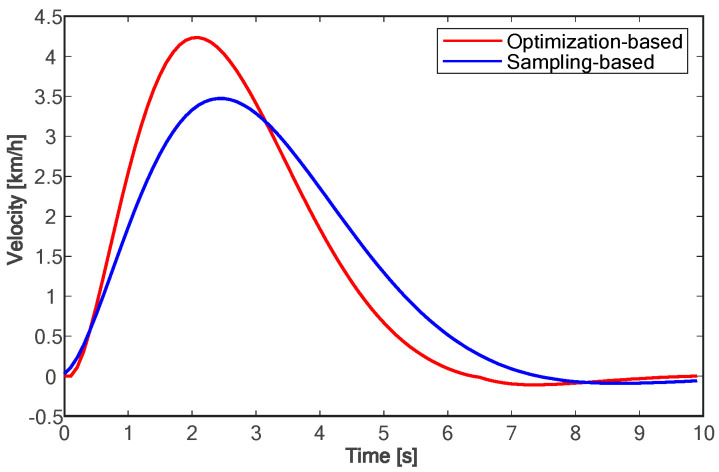
Comparison of lateral velocities.

**Figure 13:**
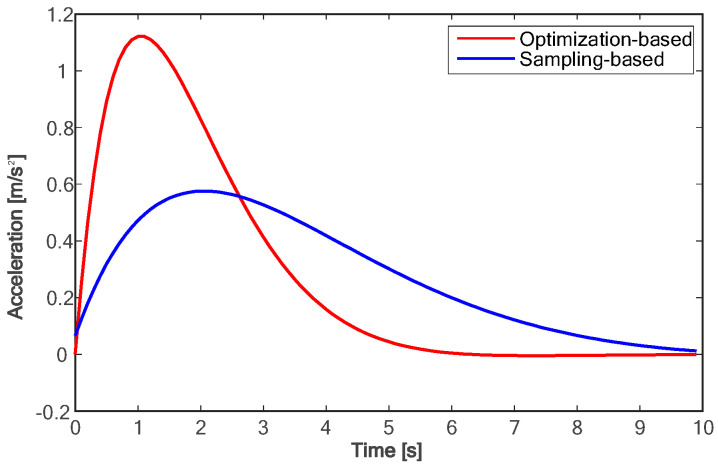
Comparison of longitudinal accelerations.

**Figure 14:**
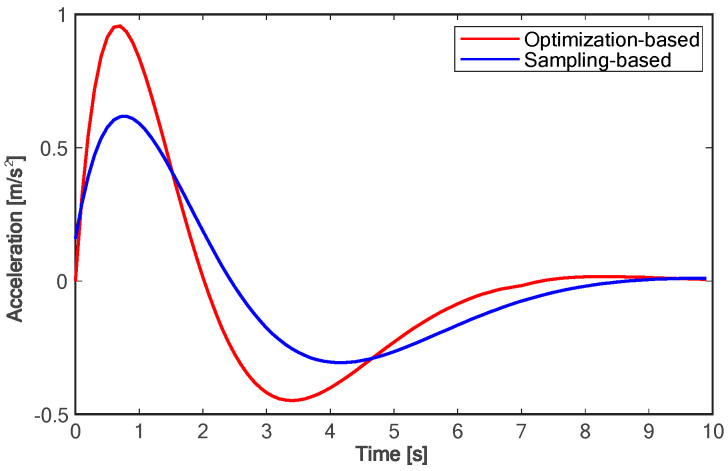
Comparison of lateral accelerations.

**Figure 15:**
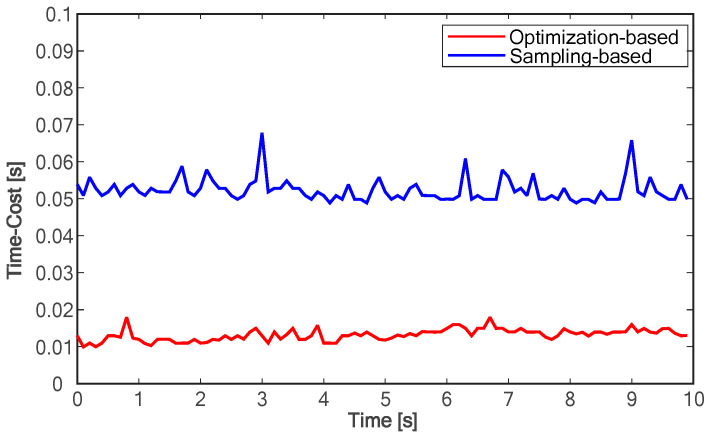
Comparison of time costs.

The proposed method is compared with the polynomial sampling method in simulation experiments. Considering that too many sampling tracks will lead to too long of a calculation time, it is difficult to ensure real-time performance. Therefore, the method based on polynomial sampling in this paper has one time sampling layer and lateral migration layer, six target velocity sampling layers, fifty control time domains, and 0.1 s sampling time, that is, the trajectory of the future 5.0 s is planned. Both approaches are implemented in Python3.11. The simulation experiment data are shown in Figure 10, Figure 11, Figure 12, Figure 13, Figure 14 and Figure 15:

Due to the fact that sampling-based methods can only constrain the terminal states of the samples, the motion parameter information for times other than the final moment in the entire trajectory is generated solely through polynomial fitting. This makes it challenging to control the motion parameters during the lane-changing process, hindering the optimal utilization of vehicle performance and meeting other requirements of the driving process as much as possible. As shown in Figure 10, compared with the method based on polynomial sampling, the method proposed in this paper has a faster lane change time of about 7 s. Furthermore, the proposed method achieves the target speed about 3 s faster than the method based on sampling (as shown in Figure 11). Figure 12 shows the variation trend of the lateral speed of the two methods over time. The time for the method in this paper to reach the maximum lateral speed is about 2 s, and the maximum speed is more than 1 km/h higher than that of the method based on sampling. As can be seen from Figure 13 and Figure 14, the proposed method can affect the acceleration and deceleration performance of the vehicle to a greater extent and reach the target state faster on the premise of ensuring the continuous acceleration of the vehicle.

In addition, in terms of calculation time, each trajectory sampled by the sampling-based method requires collision detection of obstacles, and the more sampling paths, the lower the planning efficiency, which greatly increases the calculation time. As shown in Figure 15, the calculation time of the proposed method is almost an order of magnitude lower than that of the sample-based method. The average time of the polynomial-based method per cycle is about 0.058 s, while the average time of the proposed method is less than 0.02 s.

The specific changes in major performance parameters are shown in Table 2. Compared with the sampling-based algorithm, the lane-changing duration reduced by 23%, while the time to reach the longitudinal target speed and maximum lateral speed decreased by 41% and 19%, respectively. It can be seen that the method proposed in this paper has a significant advantage in lane-changing efficiency. Additionally, the ranges of longitudinal and lateral acceleration variations increased by 95% and 63%, respectively, indicating that the proposed method can better utilize the vehicle’s acceleration and deceleration performance within the allowed range. Lastly, the average time cost decreased by 76%. Lower time cost means faster response to environmental changes. In conclusion, the optimization-based approach proposed in this paper is demonstrated to yield improvements in various aspects of performance compared to the sampling-based method.

### 6.2. On-Road Vehicle Experiments

**Figure 16:**
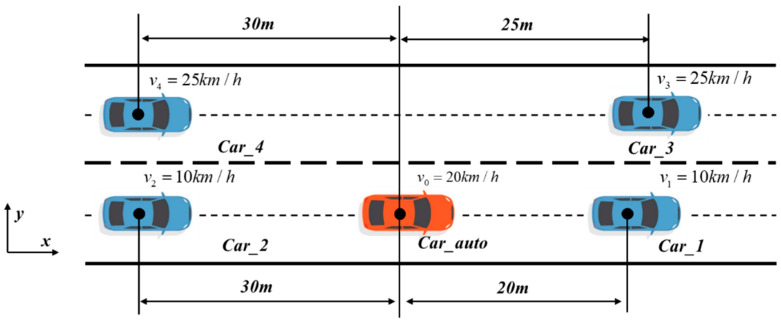
Illustration of on-road vehicle experiment scene.

In addition to simulation experiments, the performance of the method is further verified by on-road vehicle experiments. As shown in Figure 16, the on-road vehicle experiment scene shows that the ego vehicle is driving in the current lane at a speed of 20 km/h, the speed of the front vehicle and the rear vehicle in the current lane is 10 km/h, and the speed of the target lane is 25 km/h. The car needs to change lanes from the current lane to the target lane and travel at a target speed of 25 km/h.

**Figure 17:**
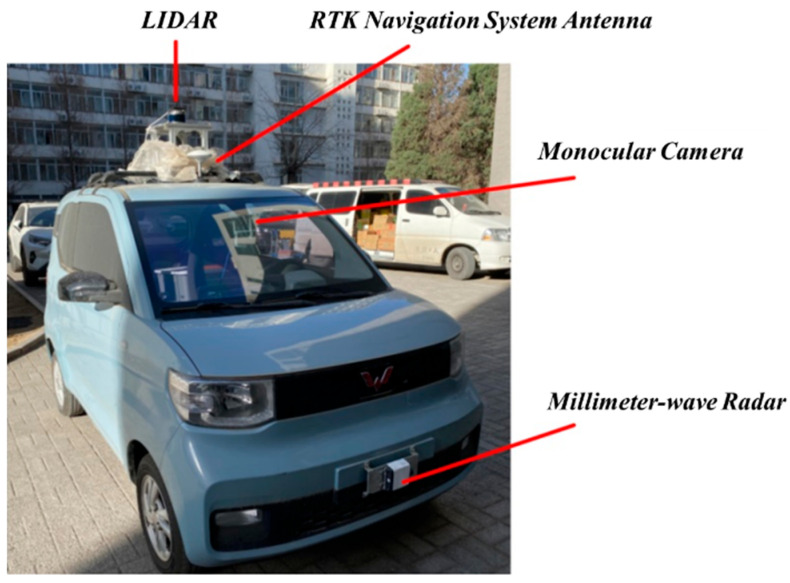
Mini EV wire-controlled platform.

The on-road vehicle experimental platform selected is the Wuling Hongguang Mini EV (SGMW, Liuzhou, China) converted to a wire-controlled platform. It is equipped with a Yanhua MIC-770-V3 (Advantech, Hongkong, China) industrial computer running the Ubuntu operating system, featuring an Intel i9-12900TE CPU (Intel, Santa Clara, CA, USA) and an RTX4070ti graphics card (NVIDIA, Santa Clara, CA, USA). Additionally, the platform is equipped with multiple sensors for environmental perception. The wire-controlled chassis facilitates the validation of various algorithms. A photograph of the actual vehicle platform is shown in Figure 17.

**Figure 18:**
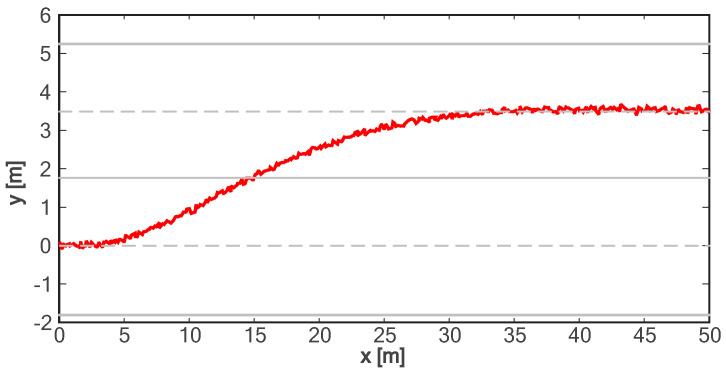
Trajectory of on-road vehicle experiment.

**Figure 19:**
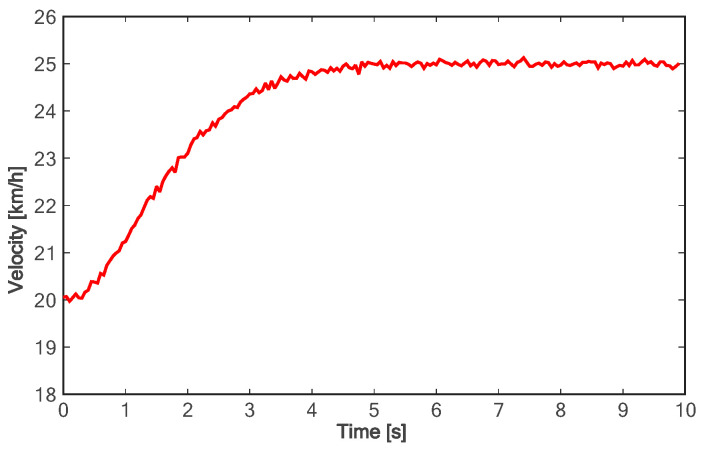
Longitudinal velocity of experiment.

**Figure 20:**
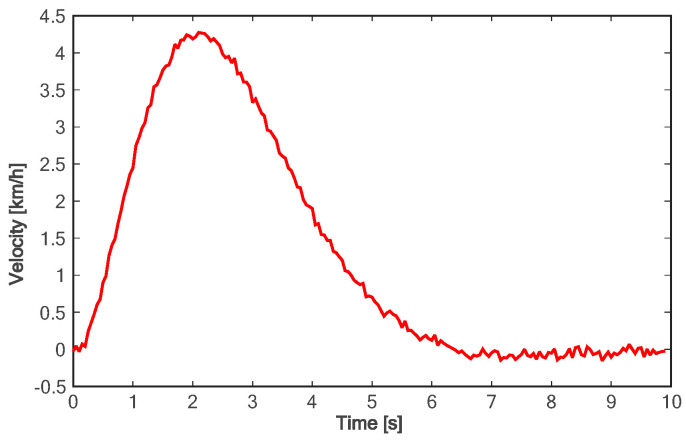
Lateral velocity of experiment.

**Figure 21:**
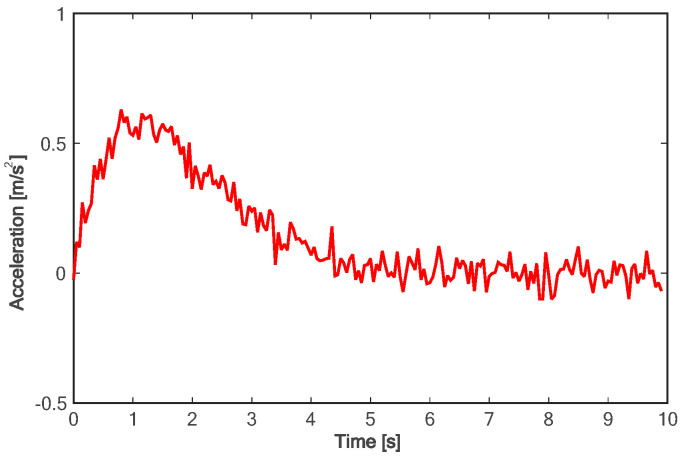
Longitudinal acceleration of experiment.

**Figure 22:**
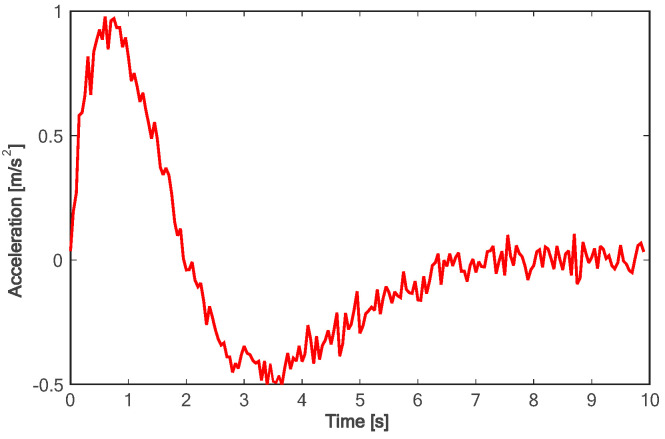
Lateral acceleration of experiment.

For the on-road vehicle experiment, the vehicle driven by the real driver is regarded as the other car, and the driver executes different driving behaviors to verify the performance of the algorithm. The self-driving car obtains the speed and position information of the other car through the on-board sensor for decision-making planning. During this period, the motion data of the vehicle were recorded and saved by the GW-NAV100B MEMS inertial/satellite integrated navigation system (Beijing Guo Wei Xing Tong Technology Co., Ltd., Beijing, China) for subsequent analysis. The parameters of the integrated navigation system are shown in Table 3. Experimental data are shown in Figure 18, Figure 19, Figure 20, Figure 21 and Figure 22.

Figure 18 shows the lane change trajectory of the vehicle in the global coordinate system. It can be seen that the lane change trajectory of the vehicle in the experiment is gentle and can be accurately converted to the designated lane. For the longitudinal acceleration process of the vehicle, it takes about 4.5 s for the vehicle to accelerate to the longitudinal target speed, and the target speed can be steadily followed (as shown in Figure 19). As can be seen from Figure 20, in the on-road vehicle experiment, it only takes about 2 s for the lateral speed to increase from 0 to the maximum value, and it decreases to zero at about 6.5 s. In other words, the lane change target is completed at about 6.5 s. As shown in Figure 21 and Figure 22, both lateral and longitudinal acceleration of the vehicle ensure smooth transition and are within the required range to ensure driving comfort.

## 7. Conclusions

In response to the autonomous lane-changing problem in complex dynamic interactive driving environments, this paper proposes an autonomous vehicle lane-changing decision-making and trajectory planning method based on graph convolutional networks (GCNs) and multi-segment polynomial curve optimization. The method constructs a GCN model for the graph-structured data of the driving environment, establishing a decision-making approach that comprehensively considers the interaction between autonomous vehicles and driving environment information. Simultaneously, the paper creates a dynamic convex polygon collision-free drivable area based on the position, velocity, and acceleration information of the ego vehicle and surrounding vehicles in the driving environment. Finally, an optimization-based multi-segment polynomial curve trajectory planning method is introduced, achieving targeted optimization of multiple trajectory segments within the collision-free drivable area.

This approach better reflects the impact of other vehicles on the autonomous lane-changing decisions of autonomous vehicles, enhances trajectory planning efficiency, and optimizes the motion parameters of polynomial trajectory curves for each segment according to the motion goal, ensuring safe and smooth motion trajectories and accurate realization of the motion goal. It addresses the limitations of completeness and generalization in traditional decision algorithms in complex dynamic environments. Experimental verification is conducted on both simulation and on-road vehicle experiments, demonstrating the feasibility of the proposed method and its superior performance compared to existing decision-making and planning methods.

In future work, this method could be extended to other scenarios such as ramp merging and intersections, and through more generalized modeling, be applied to a broader range of scenarios.

## Figures and Tables

**Table 1:** GCN construction parameters.

**Optimizer**	Adam
**Initial learning rate**	0.001
**Attenuation rate**	0.98
**Activation function**	ReLU()
**Output function**	Softmax()
**Loss function**	CrossEntropyLoss()

**Table 2:** Variations in major performance parameters.

Parameter Name	Sampling-Based	Optimization-Based	Variation
**Duration of lane change (Total)**	7.1 s	5.5 s	↓23%
**Duration to Achieve Longitudinal Target Velocity**	6.9 s	4.1 s	↓41%
**Duration to Achieve Lateral Target Velocity**	2.7 s	2.2 s	↓19%
**Range of Longitudinal Acceleration Variation**	0~0.5756 m/s^2^	0~1.1208 m/s^2^	↑95%
**Range of Lateral Acceleration Variation**	−0.3062~0.6181 m/s^2^	−0.4485~0.9565 m/s^2^	↑63%
**Average Time Cost**	0.058 s	0.014 s	↓76%

**Table 3:** Parameters of GW-NAV100B MEMS inertial/satellite integrated navigation system.

GW-NAV100B MEMS Inertial/Satellite Integrated Navigation System	
**RTK accuracy (RMS)**	Horizontal: 1.5 cm + 1 ppm; Vertical: 1.5 cm + 1 ppm
**Orientation accuracy (RMS)**	0.2°/m
**Velocity accuracy (RMS)**	0.03 m/s
**Accelerometer zero bias stability**	<0.2 mg
**Accelerometer zero bias repeatability**	<0.2 mg
**Data update frequency**	20 Hz

## Data Availability

Data are contained within the article.
